# Exploring energy citizenship in the urban heating system with the ‘Walking with Energy’ methodology

**DOI:** 10.1186/s13705-023-00393-5

**Published:** 2023-05-15

**Authors:** Jenny Palm, Aimee Ambrose

**Affiliations:** 1 International Institute for Industrial Environmental Economics (IIIEE), Lund University, P.O Box 196, 221 00 Lund, Sweden; 2 Centre for Regional Economic and Social Research (CRESR), Sheffield Hallam University, Unit 10, Science Park, Howard Street, Sheffield, S1 1WB UK

**Keywords:** Walking interviews, Energy citizens, Heating systems, Research participation

## Abstract

**Background:**

Energy citizenship has emerged as a concept which attempts to capture the new role envisaged for urban citizens as engaged and active in the energy transition. However, exactly how to successfully engage energy citizens requires more research and this article aims to contribute to this knowledge gap. The article presents a new methodology, ‘Walking with Energy’, which seeks to (re)connect citizens with where their energy is coming from. By experimenting with the application of this method in the UK and Sweden, we consider how viewing and talking about heating provision, while in the energy landscape, can encourage participants to reflect upon their local, mundane energy experiences and foster a greater sense of energy citizenship and greater motivation to engage with debates around heating transition.

**Results:**

The article presents four different events: (1) a physical walk to an energy recovery facility, (2) a walk to view a building’s heat exchanger, (3) a round-table discussion using pictures to communicate in a language café, and (4) a virtual tour around an Energy Recovery Facility. The way we conducted the events influenced who engaged, for example: the walk through a heat facility and the walk to visit a heat exchanger in the basement of a University building tended to attract white middle-class people, while the virtual tour attracted a more mixed audience in terms of age and background, but most had a strong environmental interest. The language café targeted immigrants. The different events resulted in many similar reflections, but there was also variation. For example, the walk through the heat facility generated the most focused and least diverse reflections, while the event focussed on the heat exchanger opened up a wide range of issues for discussion.

**Conclusions:**

We find that the method encouraged the sharing of personal experiences, storytelling, and deepened the engagement of participants with debates about energy. The method can help promote energy democracy and boost a deliberative dialogue about present and future energy systems among citizens. We also learnt that promotion of energy citizenship requires not only active citizens but also active facilitation to create opportunities for citizens to engage and reflect.

## Background

In 2018, the EU updated its energy policy framework to facilitate the transition away from fossil fuels to renewables. The Clean Energy for all Europeans Package [1] is part of the EU goal for an economy with net-zero greenhouse gas emissions by 2050 [2]. Within this, citizens are given a new role, and are expected to move from being passive energy consumers to active energy citizens [3]. This includes an aspiration for more decentralised and democratic energy systems and a move away from passive consumers towards a more dynamic relationship, where citizens are actively engaged with and take responsibility for everything from production and distribution to more careful energy usage [4, 5]. Energy citizenship is a key term in this future strategy and as a political, social and cultural concept it is tightly connected with an increased awareness of a need for a rapid but at the same time fair and inclusive energy transition [6, 7]. In earlier studies there are several ways in which energy citizenship has proven an important contribution to an energy transition [7]. One is by adopting and utilizing household small-scale renewable energy technologies [8] and another through participating in energy communities [9]. Participating in social movements is yet another example discussed by [8]. An alternate, more mundane understanding of energy citizenship has been influenced by studies on material engagement. People’s energy literacy will increase as a result of implementing and interacting with small-scale renewables and smart technologies in their homes, which will enable them to make connections to and act on bigger challenges [10].

However, to date, most attention in both research and policy on citizen engagement and decarbonisation has been on electricity and transport systems, with less interest in heating in the built environment. Half of the EU’s final energy consumption emanates from the heating and cooling sector, and 22% is attributed to space heating in the residential sector [11], making this an important sector for reducing fossil fuel consumption. It is the heating system that forms the focus of our research. Heating is a practice closely bound up with citizens’ everyday lives, relating to mundane yet vital activities, such as cooking and ventilation [12, 13].

Lennon et al. [14] noted that what a concept-like energy citizenship might involve in practice remains largely open to interpretation. Therefore, energy transition debates in the EU have skewed towards normative constructs of what it is to be a ‘good citizen’. Official narratives and policy cycles tend to place particular emphasis on individual behaviour change, with citizens urged to ‘play their part’ using energy more efficiently, adopting low-energy technology, and making more informed choices as consumers [14]. However, research from the field of fuel poverty has shown that system-active consumers disadvantage the less confident and capable, for example: low income households have less scope to secure the best energy deals through switching and thus effectively cross-subsidise better deals for more proactive consumers [15]. How to engage and promote parity amongst a wide range of citizens in the energy transition requires further research (see, e.g., 7, 14, 16–20).

This article presents the results from a project, where an emerging methodological concept, ‘Walking with Energy’, was used as a tool to reconnect citizens with the heating systems that have become abstract and often invisible in their daily lives and to (re)engage them with debates surrounding decarbonisation and the future of heating [21]. The method is intended to promote innovative acts of research participation, centred around actual and virtual ‘energy walks’, where the participants come together in an act of social learning to reflect upon and discuss the design and consequences of different heating systems for their lives. A key aim is to promote a greater sense of energy citizenship and motivation to engage with emerging heating transitions (whether this involves making their views known or becoming a prosumer, for example). The aim of the research is to collect data that will enable us to understand how citizens engage and interact with domestic heating systems and to capture their perceptions of environmental, political, economic, and ethical consequences of different approaches to heat generation and how these might shift as they become more informed. In this article four different events or ‘energy walks’ are analysed in relation to their content, the segments of society attracted, and the insights generated from participants.

The events were conducted in Sweden and the UK. In Sweden, the domestic heating system is dominated by district heating systems in urban settings and by heat pumps and biofueled individual heating sources in rural settings. District heating supplies 90% of heat demand in multifamily buildings, 77% of non-residential buildings, and 17% of detached and semi-detached houses. District heating supplies 58% of the heat demand [22, 23]. Electricity accounted for 26% of the heat supplied in 2016, and this figure has been stable over the past three decades. A change in the last few decades in properties off the district heating system is that most houses have replaced their electric boiler with heat pumps for improved efficiency [24]. There are over 100,000 heat pumps installed in Sweden, half of which are air-to-air pumps and one-third are ground-source heat pumps. Electricity accounts for half of the total heating demand in detached houses [22].

In contrast, the UK heating system is quite one dimensional, with 79% of homes heated through reticulated natural gas converted to heat through individual boilers in homes. The hot water is then piped around homes via a central heating system Only 210,000 homes in the UK are on district heating networks [25]. There is an increasing trend towards the installation of electric heating in new-build flats in urban areas but electric heating accounts for only ~ 7 per cent of heat provision, not least because electricity is significantly more expensive than gas in the UK [26]. Rural homes tend to be heated using liquid gas or oil, which are also expensive forms of heating relative to gas [26]. Take up of renewable energy technology for heating in the UK has been low for various reasons, the most prominent of which is the relatively low cost of natural gas and ‘lock-in’ to a gas-based system.

Before presenting the results from the events in the two countries, we will give an overview of existing literature on energy citizenship, followed by a discussion of the methods used. The four different events to engaging the public with the project are then described, analysed, and compared. The article ends with a discussion of the successes and limitations of the four events and we conclude with some considerations for policy in this area.

## Earlier research on energy citizenship

Energy citizenship is a key concept within research and debate surrounding energy transitions. It started as a critique to the generally technical focus of the transition field and its neglect of the role of democratic engagement in transition processes [7, 27]. Energy citizenship contributes to a new conceptualization of what citizenship and democracy mean in the context of energy transitions.

The concept of energy citizenship became widespread around 2010 referring to the idea that citizens have a key role to play in the energy transition away from fossil fuels [8]. Devine-Wright [28] was amongst the earliest to discuss energy citizenship and described an energy citizen as an active participant rather than a passive stakeholder in the energy system. The emergence of energy citizenship as a vital component of the energy system has later been described as an aspiration to ‘humanise’ the transition by exploring new ways of reasoning about public engagement and participation [7] and moving away from the traditional focus on energy as a technical and economic issue [7, 28].

In their literature review, Wahlund and Palm [7] gave an overview of the most common types of citizen participation discussed in relation to research on energy democracy and energy citizenship. These range from consumer choice to participation in policy processes and representative democracy. Within the energy citizenship literature, participation often refers to individual practices such as installing solar power for household use and this sort of material perspective is especially prevalent. Ryghaug et al. [10] described material participation as a mundane physical embodied experience which provides the opportunity to enact energy citizenship in new ways and take into account the actual interaction people have with technology in their everyday lives. Energy citizenship has also been put forward as a counterpoint to the social and psychological ‘detachment’ of the public from energy systems embedded within centralised energy provision systems and deficit views of energy users [14]. It can, however, be challenging to be an energy citizen and Lennon et al. [29] found in their study of five European countries that most participants did not consider themselves to have real agency in decision-making regarding their energy use other than as consumers. More narrow definitions of energy citizenship include consumer-oriented actions, such as shifting electricity consumption away from peak hours or adopting energy efficiency measures in the home [30–32]. Energy citizenship as defined within this article, will refer back to the broader definition, including the everyday interactions people have with their energy systems to the participation in the policy process.

In their study of decision-making in two community energy projects in England and Scotland van Veelen and Eadson [33] contended that ‘becoming democratic’ is a reflexive process rather than an outcome. This is in line with the views of Devine-Wright [28] when he argued that the motivation for participation is attributed to a combination of environmental concern, ascription of personal responsibility, and a desire for self-sufficiency or control. This reflexive process is also in focus for the Walking with Energy methodology developed here.

From an energy policy perspective, the motivation for involving citizens has largely been to ensure rights of access to information and transparency [7]. Another aim has been to ensure that citizens adopt new technologies when decentralising energy systems, which has been prioritised over truly involving all the relevant actors in the decision-making process and discussing options and consequences of alternative scenarios [34–36]. Thomas et al. [37] in their study of flexibility in the energy system argued that the focus on citizen engagement often fails to consider the needs of those who lack the economic and social resources to be more active in the transition by, for example, investing in technologies or adapting their practices. They mentioned the elderly, chronically ill, and people engaged in unpredictable shift work in this context. While some effort has been made to increase citizen participation in policy-making [38], developments have also been met with concern from those who fear that delegating decision-making authority to local people may jeopardise decades of hard-won regulatory success [39]. Desires for the increase of direct citizen participation in the energy system may also sit uneasily with concerns about more democratic forms of participation when structural barriers to participation are not properly addressed [7, 40].

Another challenge in relation to domestic heating, and especially district heating, relevant to this article, is that the systems suffer from ‘double invisibility’, in the sense that modern heating systems can often not be seen nor connected to everyday actions [21]. Walking interviews as a methodology have been used in earlier research to visualise the energy system, with a tacit objective to overcome this invisibility, and to reveal insights into how people experience a neighbourhood or street [41, 42]. The method encourages citizens to reflect upon an experience in the actual place it was experienced instead of doing so retrospectively and at a distance, as is traditionally the case in research [43]. In this vein, Evans and Jones highlighted how proximity and a clear line of sight to the place or feature under discussion are critical in terms of stimulating discussion, underlining the significance of first-hand encounters. Cherry et al. [44] also emphasized the advantages of situated experiences and personal exploration over abstract and technical visions, in the context of decarbonisation and socio-technical changes. Castán Broto et al. [42] heeded this lesson and applied walking methods in an urban energy context to understand relationships between citizens and energy infrastructure. The method is used to prompt participants to reflect on urban infrastructure that is vital and impacts heavily on their lives yet has become an almost invisible or taken for granted part of the local landscape [21, 45–47].

How to enhance citizen participation is, however, a challenging issue in need of more research. In the past decade, and especially during the COVID-19 pandemic, virtual citizen participation has become more attractive as information and communication technology (ICT) has developed and become more widely diffused in society. In research there are different perspectives on citizen engagement via the internet. Some contend that the internet has a negative impact on citizen involvement, because people tend to associate it with entertainment and personal communication rather than with civic activities [48]. Others argue that the internet has the potential to encourage and broaden citizen engagement [49]. However, in relation to this latter point, some counter that it will primarily reach those already empowered and engaged in politics, while others believe that internet will also mobilise the less politically active and marginalised [48].

The literature also suggests that many citizens will struggle to fulfil the role of energy citizens, via online or any other means, and it is often those who are well-educated, with moderate income, who have the time and confidence to be active and participate that will stand the best chance. It can be difficult to engage citizens, because they have multiple commitments, have other responsibilities, and can suffer from participation fatigue [50, 51].

Empowerment is a commonly used term in the energy citizenship literature and usually implies that the citizens are meaningfully involved in and take ownership of the design and development of the heating system [52]. General pre-requisites of empowerment within the energy system include higher levels of education and energy literacy, access to information and training, ownership, and capacity to imagine change etc. [53]. When empowerment is viewed as a social process, citizens’ social rights, respect, and dignity will be in the forefront, and material resources, information and knowledge will be made accessible for all [54]. Exactly how to achieve this requires further research, and this forms part of the contribution of this article.

The project, ‘Walking with Energy’ aims to promote reconnection between a demographically diverse range of citizens and the environmental and ethical debates surrounding different approaches to heating provision. The method offers participants the opportunity to visualise the energy system and even to embody it through ‘behind the scenes’ tours of power stations, encouraging reflection on their local, mundane energy experiences. It embraces the idea that energy citizenship is a reflexive process rather than an outcome. In the project, we trialled a range of different methods in pursuit of these aims, the results of which are now discussed.

## Methods

The basic premise of the project is to walk and talk with ordinary citizens and explore how they connect and interact with current heating systems and how they feel about and envisage the future transition of the system away from fossil fuels. Earlier studies using similar methods aiming to capture visual, spatial and verbal experiences from participants have proven successful when it comes to allowing the participants to express themselves in various ways and allowing for a deeper comprehension of the numerous ways they value a phenomenon [55, 56]. Carpiano [57] investigated the use of walking interviews and found that they helped to increase interviewee participation and that the method was effective in emphasizing the context of the research. Similar findings have been made by Brown and Durrheim [58] who found that walking interviews improved the understanding of the studied object, because the environment itself served as a co-producer of conversation. Evans and Jones found that walking interviews contribute to more honest answers from the interviewees, because they were less focused on giving the “right” answer to a question [41]. Problems with the method include the fact that the method can be time consuming, resource intensive and logistically challenging [59]. Yet the gains in terms of the richness and complexity of the data generated is regarded as making up for these problems.

In this project we have focused primarily on energy from waste and associated district heating networks for several reasons. First, this is a form of energy generation that connects England and Sweden in that it is an established approach to heat provision in Swedish urban areas and something the UK government aspires to roll out. It is also a fairly controversial and poorly understood (particularly in the UK) form of energy generation due to its reliance on maintaining current levels of waste generation and its links to wider debates about sustainable consumption and the circular economy and evokes controversies around definitions of what constitutes renewable and low-carbon energy sources. District heating, in particular, suffers in many ways from a ‘double invisibility’, because it is not visible in everyday life (with pipework deep underground and heat sources often located remotely) and because this system seldom demands any action from us on a day-to-day basis, unlike gas central heating systems of the type prevalent in the UK which are within the direct control of occupants, with the boiler situated within the home [21].

The invisibility of heating systems poses a challenge when approaching citizens with the aim of discussing everyday interactions with heat and future heating pathways. In the project we have combined different methods, including walking along the route of district heating pipelines, tracing them to their source at heating plants, where we take a tour. Due to COVID-19, we needed to transform the events to virtual events, which unexpectedly gave us new tools to reach a larger number and broader range of citizens to discuss heat and heating practices. The results from three physical and one virtual event and citizen’s reflections shared during them are presented here.

Over the course of the project we held twelve events, attracting a total of 206 participants. In this paper we present the results from three physical events held before the COVID-19 outbreak and one virtual walk held online. One of the physical events were in the UK and two in Sweden. The virtual events were open for anyone to attend, but one was conducted in Swedish and the other two in English. The one presented here is one of the virtual events conducted in English and led from the UK. The four events discussed here were selected to reflect the diversity of approaches we took to the events across the project. They differ in terms of how they were conducted, who participated and the issues discussed by participants. We could have chosen the events that resemble each other the most, but that would have led to another kind of analysis and the aim here was to analyse how different kind of events affected engagement, discussions and who participated.

Our approach to conducting the different events is described in more detail below. Four of the 12 events that we ran formed part of popular science programmes (see below under “How the events were conducted” for more details). This helped us to reach a larger and more diverse audience than we might have been able to achieve through advertising the events as isolated opportunities. Linking to these broader programmes gave us access to additional, structured publicity plus practical and financial support to run the events. These events turned out to be the best attended of all those we ran. Because the events operated at the confluence of a knowledge exchange event and a research exercise, purposive sampling was not appropriate and risked low levels of participation. This approach may also have required us to exclude certain groups from events which were open to the public. Instead, diverse approaches to publicity were adopted aimed at securing a broad range of participants, including the use of social media, newspaper adverts, flyers, posters and radio stations.

We fully acknowledge that self-selection bias bound up with our approach and it is likely that we attracted participants who were already enthusiastic about environmental issues. These issues are recognised and explored in the first publication to stem from this project [21]. However, it is important to note that all events were successful in attracting non-specialist audiences. Most participants engaged from a lay perspective and very few had a professional background in related areas.

Towards the end of the project, we worked to forge links with specific communities in the vicinity of power plants and developed events around their needs or integrated our project into existing events they were running. We did this to improve engagement with groups underrepresented in the project to date. Examples include: joining a language café attended by immigrant groups living in the shadow of a power station in Malmö and running workshops for elderly residents of a sheltered housing scheme in Lund which is on the district heating network. Online events necessitated by COVID-19 provided an opportunity to attract a greater proportion of younger people to our events and also to reach broader international audiences.

The same interview guide was used in all the physical events, and was aimed at a conversational style, but the online event was set up to encourage active participation, with participants’ questions central to the events. This led to different issues being raised on different events. The various events were also conducted differently, which also influenced how the participants reflected on heat and the heating system. On all events we asked the participants to give us some background information about themselves, their home, and their heating system. We asked about how they perceived a sustainable heating system and, during or after the tour, we asked for their reflections on what they had seen and whether this influenced their thinking in any way about what our future heating systems should look like. In the virtual events, we posed questions to participants that they could respond to in the chat, but the physical events were mainly based on the participants’ questions to each other and to the officials leading tours of the energy-from-waste facilities we visited. De-briefs with researchers at the end of the events provided an opportunity to reflect on what had been learnt.

During the physical events the participants were gathered in small group to discuss their experiences and reflections on what they had seen and heard and the implications for the heating transition. Deliberative methodologies where the participants are gathered in small groups to discuss has been suggested as a way to investigate ethical implications of technologies [60, 61]. Thomas et al. [60] discussed that a challenge is to avoid reproducing expert narratives and problem definitions in these situations. This has been dealt with during the events by having different perspectives present in panels and presentations to show that the heat transition can be viewed and experienced differently. The researchers leading the discussions were also instructed to facilitate the discussion in a way that encourages the presence of different perspectives and opinions, but also themselves raised questions and ideas that was lacking in the ongoing dialogue.

The discussions were either recorded or the researchers took notes that were later written up. Parts of the walks were also filmed using researchers’ mobile phones. The recorded parts were transcribed afterwards. The chat during the virtual events was saved, and we had the responses from the surveys. All participants were promised anonymity in quotations, but for some events we have the participants’ consent to use the photos taken.

## Results

We start by presenting how the different events were conducted. A comparison is then made between the different approaches and foci according to who participated, what issues the event gave rise to, and reflections shared by participants during the events.

### How the events were conducted

#### Walking through a waste facility

In Sheffield (UK) we conducted a physical walk, where the participants had a tour of Sheffield’s energy-from-waste facility. At the facility, the city’s refuse is burned to generate heat and electricity for distribution to some local households but mostly to businesses and institutions in the city centre and inner city. The walk took place pre-COVID and formed part of a nationwide programme of popular science events called the Festival of Social Science, organised by the UK Economic and Social Research Council (ESRC). Places were limited to 15 due to health and safety requirements at the energy-from-waste plant that formed part of the tour. All places were filled, and a waiting list set up due to high demand for the event.

The event began with an explanation of the aims of the Walking with Energy Project at a café on the district heating network fuelled by the plant. We then walked as a group along 1.5 km of the route of the 47 km of district heating pipeline that runs around Sheffield City Centre, distributing heat to over 200 buildings. We traced the pipeline to its source at the energy from waste facility and stopped to view major buildings supplied by the network and parts of energy infrastructure that we encountered along the route. This walk promoted embeddedness in the energy landscape and raised awareness of hidden and taken for granted energy infrastructure. On arrival at the energy-from-waste plant, we took an access-all-areas tour that took in every aspect of the process, from the arrival of waste, its incineration to produce heat and electricity, and the separation of metals from the ash stream. Participants could ask questions as they walked around and at the end of the tour. We then adjourned to a meeting room for a debrief, where participants could share their reactions to what they had seen and heard.

#### Visiting a heat exchanger in a building

One challenge with walking alongside a district heating system and through a heat generation plant is that participants need to have the strength and mobility to participate. A lighter version of the walk was tried out in Sweden during the ‘Future Week’, a popular science event organised by Lund University in 2019. During this week, we organised a walk, where the general public were invited to a University building, to listen to a presentation about Lund’s heating system and view and learn about the building’s heat exchanger. The event was marketed through the Future Week organisation, which used channels, such as newspapers, the university’s website, and social media. Participants needed to register beforehand via the Future Week website, and the number of participants was limited to 12. Ten participants registered for the event and, of these, eight attended.

The event started with a presentation of the project and the aim of the event. The participants were then divided into three groups, each with a discussion leader who was a researcher. A group discussion was initiated, where we asked the participants to introduce themselves, their living situation, how they heated their home, how they would like to heat their home, and how they perceived sustainable heating systems. Around 10 min into the talks, the first group was guided down to the basement, where a researcher demonstrated the heat exchanger and shared posters detailing the local heating system with its plants and pipes. After the tour of the basement, the group returned to their classroom for a final group discussion based on their reflections on what they had seen, whether they had learnt anything new, and whether their perceptions had changed in any way as a result of what they had learnt.

#### Language café for immigrants

In an attempt to reach a wider audience, we targeted a socio-economically challenged area in the city of Malmö, Sweden, where the city works actively with immigrants and their integration into society. The neighbourhood also hosts a large gas fired power station. We contacted the municipal area manager who distributed an invitation to different citizen representatives in the area. The idea was to meet in a community hall in the area and walk to the power plant close by. The people attending were all part of a language café for immigrants, located in the same building, and comprised five students plus their teacher. The participants had not fully understood the information about the event and were not willing to walk to the plant. The weather was bad, and they did not feel that it was safe enough to venture out. They were, however, willing to participate in a group interview to discuss the heating system in Sweden and in their country of origin.

We lacked a common language, which was a huge barrier. The teacher could not speak the students’ language but supported communication in other ways. Mobile phones also proved useful in showing what we were talking about, both from the researcher’s and participants’ side.

#### The virtual walk

The virtual walk was an online event at the same festival as the physical walk to and around the energy-from-waste facility in Sheffield, but the year after: ‘The 2020 Festival of Social Science’. The festival marketed the event, and anyone could sign up to participate. We as researchers could not control who participated.

The event started with all participants being invited to use the chat facility to state their name and where they lived. The Walking with Energy project was introduced by one of the researchers. A specially made, professionally produced film, commissioned to support the virtual walks, was then played (the film can be seen here: https://www.youtube.com/watch?v=I8_i1gU3gRg&t=42s). A waste incineration plant located in Malmö, Sweden, was filmed and the audience could follow how the heat was produced and also how the heat was transported to a family’s radiators. After the film, a panel of experts was invited to discuss the pros and cons of district heating using waste as a fuel. The audience were then invited to ask questions. They could also pose questions continually in the chat. The event lasted for 1 h and 15 min.

### Who participated

The way we conducted the events appeared to influence who attended. The walk through a heat facility required that the participants could move along the district heating pipes and through the heat facility and physically be in the energy landscape, noticing things they had not noticed before or had taken for granted. This offered a rich and novel experience but also made it difficult for people with more limited mobility to join. The attendees at this event were mainly men, educated with a professional or skilled background. The second walk, including visiting a heat exchanger in a basement, was more accessible. However, all attendees could walk and were mainly home-owners, white, middle to low income, with varied educational backgrounds. One participant was a student, aged under 30, while the others were middle-aged or older. The third walk linked to the language café, involved one teacher and five students none of them born in Sweden. Due to the language difficulties, we did not ask them about their age, but we estimated it to be between 20 and 50 years. Income and education were unknown.

The virtual walk attracted 54 participants. It was a mixed group in terms of age and background, but all are assumed to have been digitally literate. They were all interested in environmental issues, but most were not experts when it came to heat systems or how heat is produced.

The participants were those expected, with some exceptions. The majority of the participants in the heat exchange walk were home-owners with expectations of hearing about different home heating systems but also welcoming a broader discussion about heating systems in general. The virtual event attracted young people including many students.

### What issues were raised

Table 1 presents an overview of the technology and themes that were in focus during the different events.

**Table 1:** Overview of themes discussed in the different events

Event	Technology in focus	Theme discussed	
		Recurring themes	Unique themes
Walk through waste facility	Energy-from-waste facility	Emissions Waste volume and overconsumption Plastic waste Recycling—landfill Monopoly	Energy invisibility Circular economy
Heat exchanger	Waste-to-heat facilities Heat pump District heating	Heating/cooling systems Security/reliability of the system Monopoly and lock-in effects district heating (DH) Waste incineration Pricing DH	Practical issues of how the heating system works Problems occurring in the heating system Fossil vs renewables Passive house
Language café	Radiators Diesel, oil, and wood burner Solar panels	Heating/cooling systems	Different heating and cooling practices Indoor temperature Climate and heating systems Sweden vs place of birth
Virtual walk	Waste-to-heat facilities Heat pump	Emissions Waste volume and overconsumption Plastic waste Recycling—landfill Waste incineration	Different heating technology Behaviour, attitudes Energy poverty

There was some overlap in themes discussed at the different events, shown under recurring themes in Table 1. The themes in the right column are unique themes for that specific walk. The common themes discussed concerned emissions, recycling, heating systems, waste incineration, and pricing and profit associated with different heating systems. These themes were largely anticipated, but one less expected result was that the different events generated so many common themes, despite variations in the make-up of participants and the heat sources used as examples. There were some overlaps in terms of the researchers leading the events, but there was a lot of flexibility for attendees to determine the direction of the discussion.

The in person walk through the heat facility generated the most focused and least diverse reflections. This is probably explained by the direct and physical contact the participants experienced with both production and distribution systems during the walk, which perhaps kept their attention focused on issues relating to infrastructure and associated institutions. The other three events included more discussion of behaviour and heating practices, most likely because these events made greater connections to the home and home heating systems.

### Reflections from the different events

Some trends were identified in the analysis of the comments and reflections raised during the events. The walk through the waste facility via the district heating pipelines contributed to fairly consistent reactions, which involved a combination of discomfort at the volume and nature of the waste gathered from around the city and a degree of reassurance that it was being put to a ‘good use’ through being converted to heat and electricity. Examples of some reactions:

*“We’re consuming more and more, and I don’t know how you tackle that, but at least this way you can stop the waste building up and deal with it usefully and keep it out of harm’s way.”*

*“Personally, seeing all the waste is quite a sobering thing coming face to face with the consequences of our over-consumption really, isn’t it, seeing our waste pouring into a pit like that.”*

This walk challenged the invisibility of the energy system, and both the fuel and the production process became very tangible. The walk contributed to a first-hand experience of how heat is produced, and it was no real surprise that the reflections of participants centred around emissions, waste volume, and the need for a circular economy.

During the walk that included the heat exchanger, most of the reflections related back to the participants’ own home heating systems. This was probably more due to the attendees’ expectations ahead of the event, rather than the walk as such. They had expected it to be about different home heating systems, probably because the heat exchanger was mentioned in the material supporting the event. This event contributed several surprising reflections from the attendees. One example came from a participant who made the following association after she has been guided down to the basement, where both the heat exchanger and the local energy system were presented:

*“*It’s interesting, it's just that …this with all these concepts. For me, I’m not an engineer so … I do not know this… and* it’s gas and natural gas and hydropower from Holland.” (Group 3:1)*

Why she incorrectly drew the conclusion that Sweden imports hydropower from the Netherlands is unclear. Sweden does not have much gas in the system, but some still exists in the region, so gas was mentioned during the presentation, even if it was not a central topic. This, however, symbolises something important, i.e., that the walk contributed to rather free interpretations of what is seen and heard and afforded participants the possibility to integrate new knowledge from the walk with existing knowledge and past experiences.

The continuation of this quote is also interesting, because it reveals that the events contributed to social learning. This was seen in all events and also here, when the same participant continued her reasoning by saying:

*“Then he [the guide] probably misunderstood me because I don’t have electric heating … I have such a regular … what’*s it called …* it’s called water heating…” (Group 3:1)*

Another participant then jumped in and said:

*“But then you probably have an immersion heater inside it.” (Group 3:2)*

The conversation then went on between the first and second participants:

**Participant 1**: *“Yes, I don’t know, it has…”* *(Group 3:1)*

**Participant 2**: *“But you certainly have. If you don’t fire anywhere, you have it.” (Group 3:2)*

**Participant 1**: *“No, no. But it’s…it’s like a square box that stands there.” (Group 3:1)*

**Participant 2**: *“Yes, it’s electric heating.” (Group 3:2)*

The group setting encourages the sharing of personal experiences, storytelling, and the sharing of best/worst practices and lessons learned. Participants dynamically interact with each other, contributing to an engagement in the subject. The researchers inform the participants about the heating system, but the social interaction in the form of dialogue between actors is vital for the information to be interpreted and integrated into the participants’ experience.

The event linked to the Language Café was the most unique in its performance. During this event we lacked a common language, so researchers and participants used mobile phones to show each other what we meant. One of the participants showed, for example, this photo:
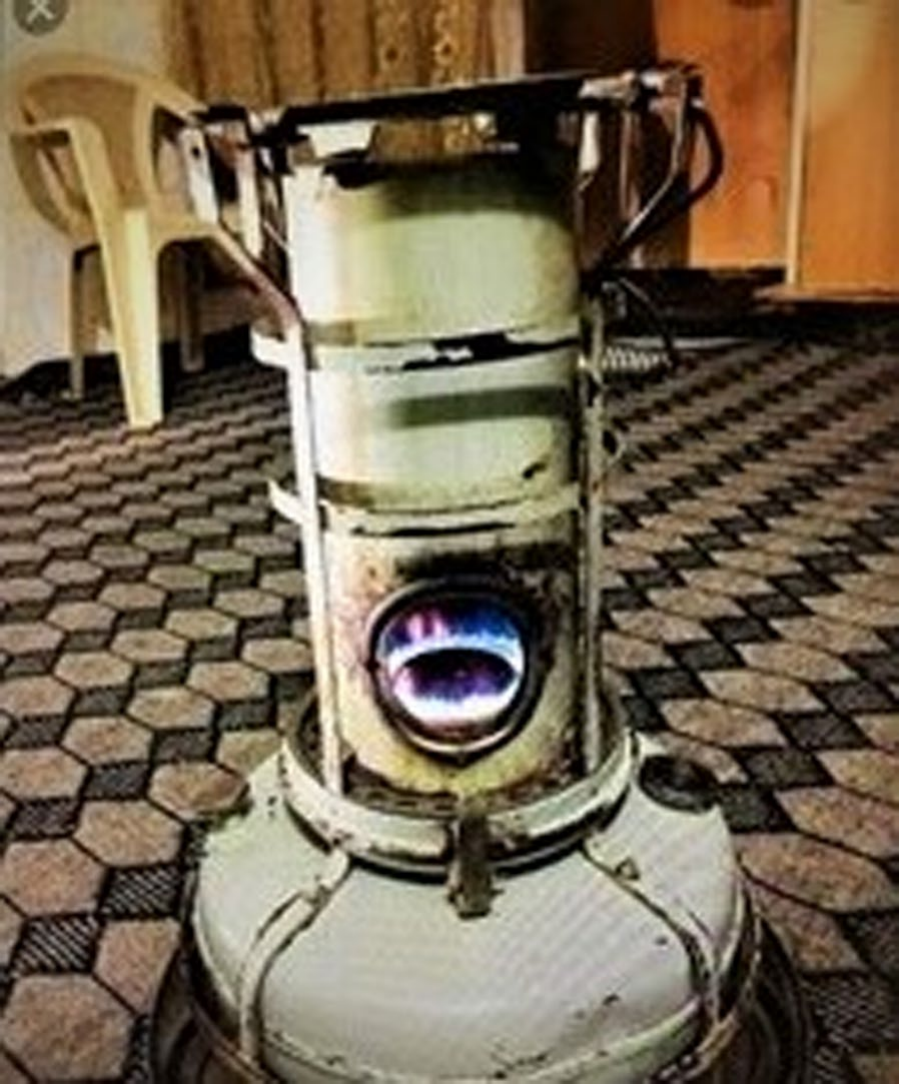


This led to information about what fuel the woman had experience of and also how the heating appliance was used:

Interviewer:* “What is this called?”*

*Participant A*:* [says a word in Arabic]*

Interviewer:* “What does that mean?”*

Teacher language café:* “Stove?”*

*Participant B*:* “Diesel and oil stove.”*

*Participant A*:* “It’s old. All the children sat around, and you make tea, coffee and food there.”*

Even when the language was restricted, we could still have an engaged discussion about the heating system. The most interesting part of this event was not what was said but what was shown, and the fact that we reached out to a group that is typically considered hard to reach in research. As researchers, we learned a lot about heating systems in these participants’ countries of birth, and we also learnt how to communicate through pictures rather than well-defined phrases.

The virtual events attracted the most participants, which is most likely explained by ease of participation. The virtual events were characterised by having two discussions running in parallel. This is a common phenomenon in virtual events, where there is often a conversation in the chat taking place at the same time as a presentation is being made. We as researchers, the invited panellists, and all the participants could contribute to the chat with reactions to what was said, general insights, and reflections. The participants could just throw out a question like this one:

*“Why do you think the waste-to-energy projects have—on average—ended up in more deprived areas in the UK? Is this something the UK government is aware of?”*

*The chat also gives possibilities to share general reflections and links, tweets, reports, blogs, etc.*,* as* this quote from the chat shows.

14:06:38* From participant A: What proportion of fossil-based waste (plastics) are in the combusted waste stream?*

14:06:46* From* participant B: what does actually happen to the ‘slag’ and other* solid end products?*

14:07:03* From* *(researcher): *https://zerowasteeurope.eu/2020/03/understanding-the-carbon-impacts-of-waste-to-energy/

14:08:19* From* *(chair of the event): @participant B—SYSAV sells metals to other recyclers and what can’t be processed goes into landfill*

14:08:53* From participant B: that’s what I thought:-(*

We did not have the time to follow up on all questions asked during the event. However, the participants had registered in advance, and we were able to send further information to everyone after the event. The active dialogue in the chat indicated that the participants were used to the virtual format and felt comfortable with such interactions. The attractiveness of online events is that they are easily accessed for those with access to information and communication technology. It is easy for everyone to see the PowerPoint presentations and follow the film compared to real-life events, where it can be hard to hear and see everything on the guided tour. One drawback is that the one-to-one chats and informal talks with the participants disappear, and everything said becomes public for all participants, is not said at all, or is shared with someone at home, unheard by the other online participants. This public nature of the discussion is positive in terms of inclusiveness and ensuring that all participants receive the same information, but it may inhibit some to contribute.

## Discussion

Earlier research has highlighted that there are different ways of understanding energy citizenship and two clear narratives dominate that literature; the top-down frame, where energy policy is believed to be the domain of experts or professional stakeholders and implemented top-down through information giving only, the bottom up frame emphasizing involvement of citizens at all stages in the policy process and through consumer choice in the market [7, 34]. In all these there is, however, a lack of attention paid to the question of *how* citizens can participate meaningfully and in a way which appeals to them thus making participation more likely and sustainable [62]. Walking with Energy in itself aims to encourage citizens to become active [28] and aware [9, 63], and empowered to take part in both the heating market and policy-making [9, 54]. The methodology emphasizes a fourth approach to energy citizenship, which involves a reflective process, recognising the importance of giving decarbonisation meaning through situated experiences and connecting it back to how heat is used and experienced in everyday life [44].

Earlier research on energy citizenship tends to overlook the importance of local community participation in debating and reflecting on existing and future energy policies. Inclusive processes, where the relationship between heat production and its use are visualised, such as walking and talking about district heating pipes and how heat is produced, can be an effective means to pursue energy democracy and energy literacy [42, 44].

The four events presented all contributed to contextualising the meaning of decarbonising the heating system. The visualisation together with a deliberative dialogue made it possible to go beyond technocratic imaginaries of the future and examine how decarbonisation was perceived by the participants and what was considered fair, desirable, accessible etc. in this context. The four events each offered different framings of the challenges connected to the decarbonisation of the heating system. The physical and the virtual events through a power plant gave rise to similar framings (e.g., spotlighting issues around emissions, plastic waste, recycling). The event at the language café and the heat exchanger enabled reflections about the heating in the homes illustrated with family histories and family dynamics. This contributed to important considerations around domestic cultures and how heating transitions can impact significantly on the mundanities of everyday life in the home. This indicates that the walk through production plants made the participants reflect upon the production of heat, while the heat exchanger and the language café focussed attention on home systems and home heating. Depending on if the domestic systems or the production process were in focus, also different parts of the transition were brought into focus. For example, consumers concern and responsibilities came to the fore during the heat exchange walk compared to the walk through the production plant, where the responsibilities of the industry were emphasized.

The language café and the visit to the heat exchanger with the personal framing of the discussions went beyond traditional issues associated with emissions and pure techno-economic aspects associated with heating provision. These events provided an opportunity to examine heating systems on a more emotional level compared to the two other events and matters of cosiness, comfort, security, and technological stress were discussed. The virtual and physical walk through a plant raised issues often acknowledged in the policy discourse around heating transitions relating to emissions, district heating as a monopoly and recycling. Reflections on more tangible issues were also more present in these events, such as over-consumption and landfilling. The physical and the virtual raised similar issues, which indicates that there might be less differences between a physical and virtual walk than we had expected. The virtual walk could not convey the sensory experience of heat production, which might give a stronger sense of, for example, the amount of waste produced by the society.

As discussed in earlier research, it is important to make citizens part of the transition process, not least because they will have to live with the consequences of it, and to re-engage them in both heating production and consumption choices that they lost touch with when we stopped burning solid fuels in the home to generate heat [21]. This reconnection appears to have been achieved to some degree in relation to all four events. The virtual and physical events resulted in a reconnection to lifestyle issues and not least over consumption and waste generation, while the language café and the heat exchanger reconnected participants to their home heating systems and the energy implications of their everyday routines. The long-term effects of the different events and the connotations they result in are yet to be established. The idea at the heart of the project was to follow up the events a year after they were conducted but due to changes in the research design necessitated by the COVID-19 outbreak, this evaluation was removed. This is, however, something we aim to pick up on in future research.

A common reflection prompted across all events was that there are many barriers to citizen participation [e.g., 50] and it has not been easy to identify and motivate people to participate in the events, and to move beyond the ‘usual suspects’ of the educated middle classes. The researchers conducting the events needed to spend much time identifying good programmes to link the events into, get access to the events, engage different heating plant operators, and not least to market the events. The events needed several researchers to lead and facilitate group interviews or the chat. Professionals were also needed to facilitate the events, such as the plant operators, to offer tours and explain the systems, and panellists to bring in different perspectives on the heating system, which prompted reflections and discussions. We can conclude that energy citizenship requires not only active citizens, but also active facilitators who give citizens the opportunities to engage and reflect on their heating experiences.

Lennon [64] called for intersectional research challenging dominant conceptions of energy and for a broader inclusion of marginalised groups. In response to this, we aimed to include a diversity of citizens and found that different citizen groups benefited from different approaches to the events (compare 65). The walks to and through a waste to energy facility appear to have appealed (although not exclusively) to those most often represented in the energy system, i.e., educated, white men with a middle-level income [21, 66]. On a conceptual level, energy citizenship includes everyone’s right to affordable energy, which in turn calls for additional participation mechanisms and the right of citizens to participate in decision-making processes [7]. However, to reach more marginalised groups, such as the immigrants targeted through the language cafe, other approaches and tools for communication might be needed, particularly given the precarity and complexity that many marginalised groups experience in daily life, which render indulging curiosities and research participation a low priority. Drawing out the explicit relevance of the events to important everyday tasks and struggles (i.e., energy affordability), may help make these types of events feel more relevant to those leading complex lives.

In our study we encountered challenges in engaging immigrants and found we needed to expressly seek them out and to work through intermediaries to secure their participation. The language café did not contribute in-depth research data to analyse, but represented success in terms of engagement of a group that is quite invisible otherwise both in research and in policy [14, 54].

The events were successful in enabling a form of social learning [46]. This was also possible through the online events, but to a lesser extent. The group setting encourages the easy exchange of personal experiences, storytelling, and best/worst practices and lessons learned. Participants dynamically interact with each other, exchanging knowledge and contributing to a deeper and more personal engagement in the subject. One final reflection is that the participants came to the events with different expectations. The participants visiting a heat exchanger expected it to be a walk focusing on how to maintain a home system. The expectations of the participants at the language café seemed mainly to be to have a chance to speak Swedish and increase their knowledge about Swedish society. In both these cases their expectations could partly be met as a result of the fact that participants were to a large extent in control of the direction of the discussions and were free to ask questions. This supports the need to have a method which give prominence to the participants experiences rather than prescribed themes or issues. At the same time it was fortunate that the different groups did not diverge too much in their expectations, otherwise this may have posed a challenge in terms of ensuring all participants felt acknowledged and included. All events were preceded by written descriptions of the walk, which most likely contributed to setting the expectations of participants. In retrospect, it might have been useful to have a control group reviewing the written invitation. In the virtual and physical tours it seemed like the participants expectations were met.

## Conclusions

Energy citizenship is strongly connected to energy democracy. An inclusive approach benefits energy transitions by increasing social acceptability of the changes required, involving usually marginalised groups and facilitating effective policy implementation. The practical and moral benefits of meaningful citizen engagement are not sufficiently heeded in relation to energy policy and need further attention and how such participation should be designed and enabled is disputed in policy and research. Walking with Energy is pursuing energy citizenship as a reflexive process rather than an outcome and our research implies that this is an important aspect of energy citizenship which contributes to engaging citizens in a deliberative dialogue on heat transition. Heating is a vital part of everyday life, and how future more sustainable systems are designed concerns all citizens. All citizens need to be included in a deliberative dialogue related to consumption and how it is connected to processes of production which profoundly affect the environment.

Another important finding of central concern for policy makers is that energy citizenship requires not only active citizens, but also active facilitation that gives citizens opportunities to engage and reflect on their heating experiences and hopes for the future. It is important for policymakers to assign both responsibility and resources to facilitating and enabling energy citizenship. Arenas for energy citizenship need staff and resources to identify and motivate people to participate, to find good public events to join as a direct route to reach citizens, for marketing events and to engage panellists and heating plants operators who can guide and explain the system.

A methodology such as the Walking with Energy contributes to empowering individuals to become more aware of heat production and consumption at a time of transition and to form views on how the future heating system should look and function. As a reflexive process it can contribute to an increased awareness of the role of energy production in climate change among the public, spark an interest in knowing more about the heating system in the home and waste incineration and promote well-informed views.

Some questions were also raised that require further research. The virtual events allowed us to accommodate a larger number of participants and increased the number of young participants in our events. However, in the main, the participants attracted were quite mainstream in terms of levels of education and professional background and more research is needed on how to include other groups. Few earlier studies have considered people’s willingness to engage with energy issues. Due to both a psychological and social “detachment” from energy many people might not be equipped with the knowledge they need to comprehend the opportunities for involvement or the motivations behind engaging with energy issues. People are not looking out for opportunities to engage more in the energy system, which most likely also affect the participation in events like ours. Further research is needed to establish how to attract and engage people in general and also a more diverse audience. Another important issue is whether these kinds of events can foster lasting increases in energy literacy and empowerment. This is an important issue that needs a longer time-period over which to study the effects, but also tools to measure an increase in less tangible values connected to energy citizenship, such as reflexiveness and engagement. These tools need to be further researched, as does the potential of participatory and reflexive approaches, such as Walking with Energy to effect lasting changes in energy literacy and citizenship. The experimental research we have undertaken here suggests there is significant potential to make energy feel more relevant to a wide range of citizens and to rediscover its centrality to our everyday lives, but how long does this effect last? And what actions do participants take away from their participation, if any? These questions require a more structured approach to the study of the potential of Walking with Energy and similar initiatives, which track the impact of participation at regular intervals following participation.

## Data Availability

The data sets used and/or analysed during the current study are available from the corresponding author on reasonable request.
